# Successful Reuse of a Liver Graft in Transplantation: First Reported Case in Chile

**DOI:** 10.7759/cureus.79136

**Published:** 2025-02-17

**Authors:** Valeria Galaz, Camila Sotomayor, Matías Orellana, Rolando Rebolledo, Julio Benítez, Erwin Buckel

**Affiliations:** 1 Department of Liver Transplant, Hepatobiliary, and Pancreatic Surgery, Hospital Dr. Sótero del Río, Santiago, CHL; 2 Department of Hepatobiliary and Pancreatic Surgery, Pontificia Universidad Católica de Chile, Santiago, CHL

**Keywords:** hepatic transplant, liver graft reuse, liver transplant, liver transplant physician, reuse of a hepatic graft

## Abstract

Organ donation in Chile remains low, creating a significant shortage of organs for transplantation and leading to long waiting times. A considerable proportion of patients on the liver transplant waiting list do not receive a transplant, often due to clinical deterioration or death.

Several strategies exist to expand the organ pool for liver transplantation. The most commonly used approaches include living-donor liver transplantation, splitting a deceased donor liver into two grafts, and utilizing marginal donor grafts. Less frequently employed methods include donation after circulatory death (DCD), domino transplantation, and the reuse of previously transplanted livers. It is also crucial to optimize their utilization by carefully matching them with lower-risk recipients.

There are some case reports and small series of cases regarding the successful reuse of liver grafts in the early and late post-transplant periods.

We report the first case of the reuse of a deceased donor liver graft in Chile. A 51-year-old woman with fulminant liver failure initially received the graft, which was later donated following her brain death caused by an intracerebral hemorrhage. After undergoing two additional hours of ex situ hypothermic oxygenated perfusion (HOPE), the graft was successfully transplanted into a 55-year-old man with autoimmune hepatitis. The recipient recovered without complications.

## Introduction

Although the number of deceased organ donors has increased in recent years, the gap between the number of patients on the waiting list and the availability of donors has continued to widen.

Organ donation in Chile is reported at a rate of 8.8 per 100,000 inhabitants, resulting in a significant shortage of organs for transplant teams and long waiting lists. The dropout rate from the liver transplant waiting list in Chile is approximately 40%, defined as removal from the list due to death or clinical deterioration [1].

According to the national transplant registry report published by the Public Health Institute of Chile, in 2023, there were 407 potential recipients on the liver transplant waiting list, but only 189 patients underwent a liver transplant. Of these, 26 were living-donor liver transplants [1].

The shortage of donor organs has compelled transplant centers to use organs from nonstandard donors [2,3].

The reuse of a graft previously transplanted into another recipient could be a viable option in this context. However, due to the susceptibility of liver grafts to ischemic injury, such reuse requires meticulous consideration and evaluation [2].

Some case reports [3-7], a small series of cases [2,8,9], and a single systematic review [10] on the reuse of liver grafts in both early and late post-transplant periods have been published internationally; however, no national reports on this topic exist in Chile. In fact, to the best of our knowledge, there is only one reported case in South America [4].

Given the lack of local information on this topic, we decided to report the first case of liver graft reuse in Chile.

All data were collected retrospectively through electronic medical records after obtaining permission from the Institutional Review Board (IRB). Informed consent was waived given the retrospective design of the study. Transplant surgeons who plan to reuse hepatic grafts for their patients may benefit from our report.

## Case presentation

A 51-year-old female patient was admitted to the critical care unit with a diagnosis of fulminant liver failure. According to King's College Criteria of non-paracetamol acute liver failure, the patient had an international normalized ratio (INR) of >6.5 upon arrival to the hospital and also had been intubated for hepatic encephalopathy previous to transfer.

After two days on the transplant waiting list, she received a liver graft from a 33-year-old deceased female donor from a local hospital. The definitive biopsy of the liver graft during procurement revealed 10% macrosteatosis. The graft underwent three hours and 22 minutes of ischemic time before being connected to oxygenated perfusion. It then underwent two hours and 19 minutes of ex situ hypothermic oxygenated perfusion (HOPE) prior to implantation using the piggyback technique. The procedure was completed without post-reperfusion syndrome, with an estimated intraoperative blood loss of 3,000 mL and norepinephrine requirements reaching up to 0.12 mcg/kg/minute. The patient received a transfusion of eight units of packed red blood cells, along with the reinfusion of 300 mL of autologous blood collected using the Cellsaver (CS) device (Sorin Xtra® Autotransfusion System, LivaNova, London, United Kingdom). Peak transaminase levels at six hours post-transplant were aspartate transaminase (AST) of 526 and alanine transaminase (ALT) of 218. Detailed post-transplant laboratory tests are shown in Table 1.

**Table 1:** Detailed post-transplant laboratory tests of the first recipient AST, aspartate transaminase; ALT, alanine transaminase; INR, international normalized ratio

Posttransplant laboratory tests	6 hours	12 hours	24 hours	48 hours	72 hours	Normal value
Lactate (mmol/L)	4.6	1.6	0.7	0.8	1.3	0.5-2.2
Bilirubin (mg/dL)	6.61	5.39	4.16	4.2	2.98	0.2-1.2
AST (U/L)	526	445	404	478	420	5-34
ALT (U/L)	197	180	176	215	218	0-55
Creatinine (mg/dL)	-	1.29	-	1.69	1.79	0.57-1.11
INR	1.77	1.46	1.5	1.2	1.1	0.97-1.27
Platelets (thousand/µL)	44	32	28	16	21	150-450

**Figure 1:**
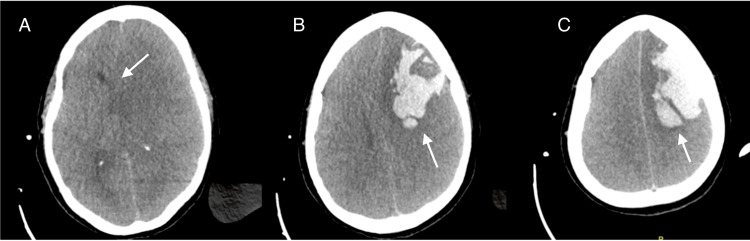
First recipient brain CT scan on day 2 after transplant (A) Arrow: approximately 9 mm of midline shift to the right, left transtentorial herniation, and apparent compression of the brainstem against the cerebellar tentorium on the right side. (B) Arrow: extensive left frontal lobar hematoma measuring at least 45 cc. (C) Arrow: another CT scan slice showing the same left frontal lobar hematoma

At 36 hours postoperatively, a new finding on physical examination revealed nonreactive mydriatic pupils. A brain CT scan showed an extensive left frontal lobar hematoma measuring at least 45 cc, with approximately 9 mm of midline shift to the right, left transtentorial herniation, and apparent compression of the brainstem against the cerebellar tentorium on the right side (Figure 1).

On postoperative day 4, brain death was confirmed according to the national protocol criteria with a complete neurological examination, an apnea test, and the patient's family expressed their intention to donate her organs.

The liver graft, which had been implanted four days earlier, was functioning adequately, as shown in Table 1, which demonstrates a sustained decrease in bilirubin and INR, as well as low transaminase levels.

It is important to note that, following the diagnosis of intracerebral hemorrhage in the first recipient at 36 hours post-transplant, an increase in lactate levels, transaminases, and bilirubin was observed (see values at 48 hours post-transplant in Table 1) after a previously downward trend. We interpret this finding as being related to the fact that, after the diagnosis of brain death but before obtaining family approval for organ donation and considering the absence of prior cases of graft reuse in our country, the patient was initially managed as a non-donor. In this context, certain treatments were limited, including even the suspension of corticosteroids for one day.

**Figure 2:**
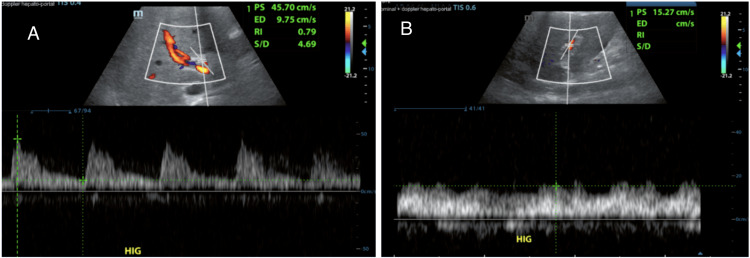
Doppler ultrasound (A) Hepatic artery at the hilum. (B) Main portal vein

Additionally, a Doppler ultrasound (Figure 2) confirmed a permeable main portal vein, with hepatopetal flow (toward the liver) and preserved Doppler spectral curves. It reaches velocities close to 66 cm/second. The hepatic artery at the hilum was observed to be permeable with velocities reaching approximately 52 cm/second and a resistive index of up to 0.79. Permeable intraparenchymal branches on the right and left were observed, with normal morphology and resistive index between 0.63 and 0.7. Permeable suprahepatic veins with hepatofugal flow and triphasic spectral waveforms were observed.

**Figure 3:**
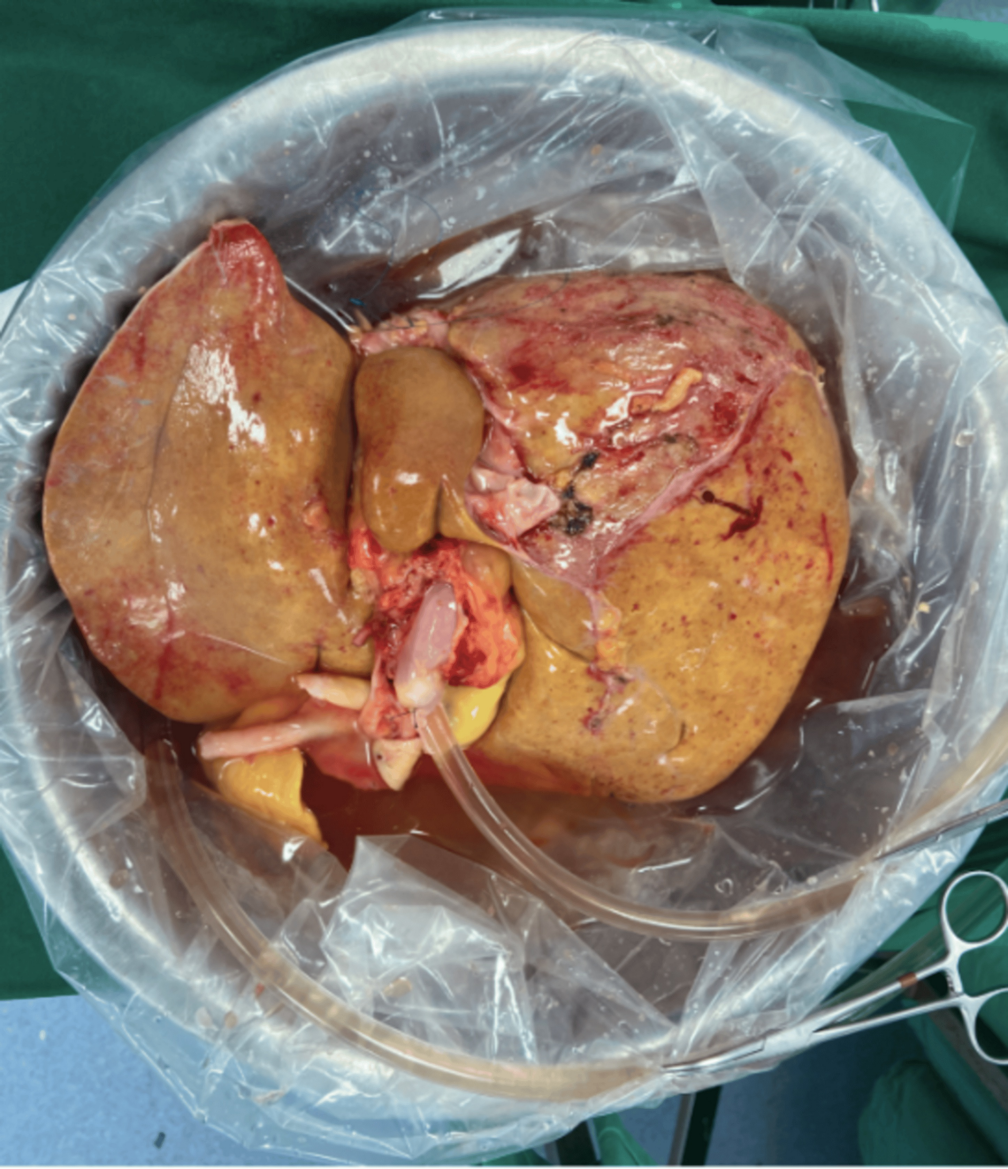
Liver graft during HOPE before the second implant HOPE: hypothermic oxygenated perfusion

**Figure 4:**
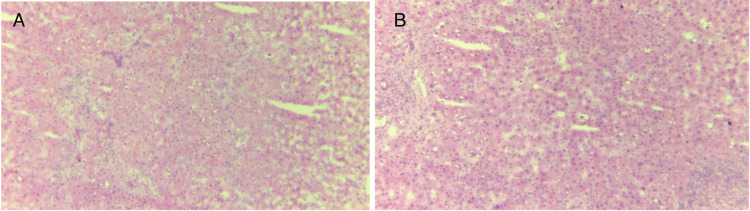
Graft biopsy during the second procurement (A) First sample. (B) Second sample

During the second procurement, the graft experienced an additional two hours and 18 minutes of cold ischemia and then underwent four hours and 52 minutes of HOPE prior to implantation (Figure 3). A biopsy obtained during the second procurement revealed 20% microvesicular steatosis, no necrosis, and moderate portal hepatitis (Figure 4). Macrosteatosis was also assessed by a specialized pathologist at the time of procurement, showing 0% steatosis at this time.

Following the second procurement, the liver graft was implanted in a 55-year-old male patient with chronic liver disease secondary to autoimmune hepatitis and a model for end-stage liver disease sodium (MELD-Na) score of 16. He had been on the waiting list for nine months and had a history of variceal bleeding, hepatorenal syndrome under medical management, and severe ascites requiring repeated paracentesis. Considering the patient's age, nutritional status, performance status, minimal sarcopenia, and relatively low MELD score, the Enlistment Committee approved the use of an extended criteria graft for this patient.

The cold ischemic time of the graft until the connection to HOPE was two hours and 18 minutes; then, HOPE was received at two hours and 52 minutes. The procedure utilized the classic piggyback technique, classic end-to-end portal vein anastomosis, and arterial anastomosis between the proper hepatic artery from both graft and receptor with 7-0 Prolene interrupted suture resulting in a warm ischemic time of 26 minutes. The biliodigestive anastomosis was done with choledochoduodenum technique. There was no post-reperfusion syndrome, intraoperative blood loss was 3,500 mL, and vasopressors were required, specifically norepinephrine at up to 0.2 mcg/kg/minute, which was discontinued six hours postoperatively. The patient received a transfusion of seven units of packed red blood cells, along with the reinfusion of 1,150 mL of autologous blood collected using the CS device.

The patient recovered without complications and was discharged from the intensive care unit on postoperative day 4, only remaining with acute kidney injury stage 2 based on Kidney Disease Improving Global Outcomes (AKI KDIGO II), which recovered without the need for dialysis, and was home on postoperative day 9, following immunosuppressive therapy adjustments until standard tacrolimus plasmatic levels were reached. Additional prednisone and later mycophenolate were added to immunosuppressive therapy.

Table 2 shows the evolution of postoperative laboratory tests of the second recipient.

**Table 2:** Postoperative laboratory tests of the second recipient AST, aspartate transaminase; ALT, alanine transaminase; INR, international normalized ratio

Post-transplant laboratory tests	6 hours	12 hours	24 hours	48 hours	72 hours	7 days	10 days	Normal value
Lactate (mmol/L)	1.8	1.2	1.2	1.1	0.8	0.9	-	0.5-2.2
Bilirubin (mg/dL)	7.4	6.9	5	4.7	3	1.8	2	0.2-1.2
AST (U/L)	534	415	241	134	68	43	63	5-34
ALT (U/L)	124	121	102	90	70	51	73	0-55
Creatinine (mg/dL)	1.6	2.4	2.9	3.3	3	-	0.7	0.57-1.11
INR	1.6	1.2	1.3	1.2	1	1	0.93	0.97-1.27
Platelets (thousand/µL)	73	61	45	52	23	24	50	150-450

To date, it has been seven months since the liver transplant, with bimonthly medical visits, and no graft-related complications have been identified.

## Discussion

The increasing disparity between organ donation and the demand for organs requires the use of unconventional donors to increase organ availability. This disparity is likewise observed in Chile and other South American countries [4].

The shortage of donors in relation to patients on the waiting list requires the consideration of any possible organ donor in order to meet the current demand, as well as the adoption of several strategies to expand the organ pool [3]. In this context, the reuse of a liver graft was first reported by Moreno et al. in 1991 [11].

Since that date, some case reports, a small series of cases, and, to the best of our knowledge, a single systematic review regarding the reuse of liver grafts have been published. In most cases, reused liver grafts have been retrieved from deceased donors who underwent transplantation shortly before reuse, in the majority of cases, within one week after transplantation, typically in the setting of acute liver failure where the recipient experienced neurological complications leading to death, as observed in this case [12]. These cases may represent a potential source of grafts not only for livers but also for other organs. Rare case reports exist of liver grafts being reused years after the initial transplant [2,8,10,12]. Notably, long-term survival rates of recipients of reused liver grafts have been reported to be comparable to those of recipients of primary liver transplants [6,8].

Even less common are reports of the reuse of living-donor liver grafts in a second recipient [6]. Severe brain damage in liver transplant recipients can result from various causes, some of which may create circumstances that allow for the reuse of liver grafts [13,14]. According to the literature, cerebrovascular accidents and cerebral edema are the most common causes of death in donors of previously transplanted grafts. Brain death due to cerebral edema is of particular concern in candidates with fulminant liver failure. Coagulation dysfunction, hemodynamic instability during the immediate postoperative period, and hypertensive crises may contribute to the occurrence of severe CNS hemorrhage with fatal outcomes following liver transplantation, as was the case with the first recipient of our graft [13].

Moreno González et al. identified several key factors for the successful reuse of liver grafts: grafts should originate from young and stable initial donors, demonstrate excellent function in the first recipient (this response is the best safety sign, given that the second implant reproduces the activity shown previously so long as the duration of warm and cold ischemia is minimal and the reperfusion damage is practically nonexistent), and undergo early reuse (within 48 hours) with minimal preservation times [15]. Additionally, there must be a negative donor-recipient crossmatch, ABO compatibility, and the absence of viral, bacterial, or fungal infections.

Regarding the evaluation prior to graft reuse, a systematic review [10] and a case series [8] have proposed performing a routine biopsy before the second transplant to confirm minimal preservation injury and predict the risk of primary graft non-function. However, another study suggests that non-functional status seems quite non-predictable [2].

Laboratory findings appear to hold greater significance than biopsy results. According to certain international transplant groups, a total bilirubin level of 3 mg/dL or higher in the donor may render the graft unsuitable for transplantation [2].

Although caution is essential, the current report suggests that the criteria for donation following transplantation may closely align with those for conventional donation after neurological death.

The clinical indications for liver graft reuse vary across reported cases, with conditions such as hepatocellular carcinoma (HCC), chronic rejection, and recurrent hepatitis suggesting that candidates may have been considered for this unconventional approach due to limited access to the standard transplant waiting list [10]. Also, the possibility of reusing liver grafts may be considered when an urgent transplant for patients with terminal hepatic failure is required and there are no available donors [15].

Currently, there are no established guidelines for determining recipient eligibility for reused liver transplantation. However, this option may benefit marginal recipients whose general condition is declining, whose malignancy is approaching the threshold for transplant eligibility, and for whom a suitable donor is unavailable.

Another factor to consider is that, since June 2016, liver graft allocation has been based on the MELD system. A higher MELD score indicates a more critical pretransplant condition, subjecting the hepatic graft to greater physiological stress. In such cases, the reuse of a hepatic graft would require increased caution [2].

It remains uncertain whether the reuse of a liver graft increases its vulnerability to ischemia/reperfusion injury, potentially resulting in a higher degree of steatosis or poorer outcomes compared to standard deceased donor grafts. Nonetheless, until further experience with reused liver grafts is accumulated, we agree with the recommendations made by other groups to classify them as extended criteria donor grafts, acknowledging their potential limitations, particularly for transplantation in patients with a MELD score exceeding 25 [8]. Our second recipient had a MELD-Na score of 16, making him an appropriate candidate.

Regarding the surgical technique, It is important to highlight that the repeated transplantation of a single liver graft in two different recipients may represent a technical challenge and thus increase the risk for the recipient [8].

First, the previously constructed vascular anastomoses must be identified during the donor operation and meticulously dissected during the cold preparation phase. This step ensures the accurate adjustment of the portal vein length during implantation, with the intentional resection of the primary anastomosis to prevent segmental stenosis [8].

Second, in our case, we performed the piggyback technique for the cavocavostomy (a side-to-side cavocavostomy).

The surgical techniques for organ implantation in the setting of liver reuse are variable, especially with respect to the management of venous drainage; they range from the piggyback technique to the full replacement of the recipient's inferior vena cava, the latter being the most commonly used, although a laterolateral cavocaval anastomosis could be performed as well. We chose to use the classic piggyback technique (the preservation of the recipient vena cava) and include the previous anastomosis because the previous transplant was recent, so the vascular structures could be clearly identified. The present vena cava anastomosis consisted of three layers of venous tissues (two donors and the recipient). Also, this is our preferred technique because it shortens the warm ischemia time.

Arterial reconstruction is typically not a significant challenge, as the graft itself generally provides adequate arterial length. Alternatively, the celiac trunk from the first recipient (now acting as the donor) can be utilized. In cases where the anatomical configuration or the arterial supply to the graft is compromised, an interposition "jump" graft between the aorta and the hepatic artery of the graft, using donor-derived iliac vessels, can be employed as a viable solution [8].

Biliary reconstruction remains a critical challenge in liver transplantation, especially when reusing a liver graft. In such cases, the previous biliary anastomosis from the initial transplant is often encased in scar tissue, raising concerns regarding the adequacy of the local blood supply. Thus, to avoid the segmental ischemia of the biliary reconstruction, connective tissue surrounding the biliary tract was spared from dissection. In the case of our patient, we performed a bilioenteric anastomosis, which we considered the safest option for reconstruction in this particular case.

In addition to technical challenges, the impact of chronic immunosuppression on the function of reused liver grafts remains an unresolved issue. In our case, histological analysis showed no evidence of chronic vascular changes associated with calcineurin inhibitors. Long-term toxic effects of immunosuppressants in liver transplantation are rarely reported in the literature, and calcineurin inhibitor-induced vasculopathy appears to be uncommon in liver transplants, unlike in kidney transplants. It has been suggested that chronic exposure to tacrolimus may provide some protection against ischemia and reperfusion-related signaling pathways that compromise graft survival, potentially reducing cell death and contributing to improved graft function, which could be particularly significant in cases of the late reuse of liver allografts [8,16,17].

Currently, transplant programs are increasingly considering marginal donors, including older donors, donors with fatty liver, or those with other conditions, despite the higher likelihood of delayed graft function or suboptimal outcomes compared to non-marginal donors. In patients with limited options, reuse can be carefully considered after thoroughly assessing the graft for ischemic damage and the status of the recipient. Ideally, the local ethics committee should be consulted before proceeding with the reuse of a liver graft.

This detailed documentation of a case of liver graft reuse for a second organ recipient supports the conclusions of different international groups: this strategy is definitely feasible.

However, although the majority of reported cases show favorable outcomes, the global experience with reused liver grafts is still too limited to generalize these results. We hope that our report provides valuable guidance for liver transplant surgeons and their teams when confronted with exceptional situations, such as the unexpected death of a recipient posttransplantation in circumstances that allow for organ donation.

Considering all the above, an individualized approach to organ acceptance and surgical technique is recommended, especially in liver transplantation. This is of great importance as an alternative for patients at high risk of dropout who are lower on the waiting list.

Specific challenges in Chile regarding the reuse of liver grafts stem from a lack of awareness among procurement and transplant teams. Post-liver transplant patients are often perceived as critically ill, and when death occurs due to causes such as intracranial hemorrhage, they are rarely considered potential organ donors, despite the possibility of near-normal graft function. Liver transplant teams are ethically restricted from influencing donor selection, further limiting these opportunities. Additionally, transplant teams may be reluctant to use a liver graft that has already undergone ischemic injury, particularly in the absence of preservation with perfusion techniques. This is the case for most transplant centers in Chile, as only two centers in the country currently perform oxygenated perfusion techniques.

Organ donation rates in Chile remain low, primarily due to a lack of awareness about the process. Although organ donation is mandated by law, most families oppose it, and their wishes are respected. However, families of liver transplant recipients, who are more familiar with the transplantation process and its challenges, may be more inclined to consider donation following a neurological complication after transplant.

This case report aims to raise awareness among procurement teams regarding the potential for liver transplant recipients to become donors, thereby contributing to the expansion of the donor pool.

## Conclusions

This report presents the first documented case in Chile of the early reuse of a liver graft, highlighting its potential to expand the donor pool and address organ shortages. Reusing a previously functional graft allows for resource optimization, maximizing the utility of available organs while ensuring effective allocation. Understanding the graft's history, including its prior function and immunological response, aids in predicting post-transplant performance and improving patient outcomes. Additionally, this practice may contribute to reducing waiting times for recipients lower on the transplant list.

The literature suggests several key criteria for liver graft reuse: The initial donor should be young and hemodynamically stable, the graft must have demonstrated optimal function in the first recipient, and short preservation times, minimal preservation injury confirmed by biopsy, immunological compatibility, and the absence of infections are also critical considerations. This case demonstrates that, when these conditions are met, the reuse of a marginal graft in an appropriate recipient can lead to successful transplantation, with no postoperative complications, a short hospital stay, and timely organ allocation.

## References

[1] (2023). Registro nacional de trasplante 2023. https://www.ispch.cl/wp-content/uploads/2024/10/Registro-Nacional-de-Trasplante-Informe-2023-final.pdf.

[2] Kim HY, Choi B, Kim M, Choi Y, Lee J, Cho WH (2021). Reusing hepatic grafts in Korea: a case report. Korean J Transplant.

[3] Karabulut K, Eris C, Piskin T, Kayaalp C, Yilmaz S (2012). Reuse of a pediatric liver graft: a case report. Case Rep Transplant.

[4] Balderramo D, Eugenia Romero M, Alcaraz Á, Barrabino M, Maraschio M (2015). Reuse of a transplanted liver graft: first experience in South America. Liver Transpl.

[5] Kim MJ, Hwang S, Jung DH, Park GC, Song GW, Cho HD, Lee SG (2020). Reuse of liver allograft from a brain-dead recipient: a case report. Ann Hepatobiliary Pancreat Surg.

[6] Hu XG, Kim IG, Wang HJ, Kim BW, Hong SY, Kim YB, Shen XY (2018). Reuse of living-donor liver graft in second recipient with long-term survival. Transplant Proc.

[7] Nafidi O, Letourneau R, Willems BE, Lapointe RW (2007). Reuse of liver graft from a brain dead recipient. Clin Transplant.

[8] Rentsch M, Meyer J, Andrassy J (2010). Late reuse of liver allografts from brain-dead graft recipients: the Munich experience and a review of the literature. Liver Transpl.

[9] Castellote J, Lladó L, Xiol X (2006). Successful reuse of liver grafts after death of the first recipient. Clin Transplant.

[10] Tanaka H, McAlister VC, Levstik MA, Ghent CN, Marotta PJ, Quan D, Wall WJ (2014). Reuse of liver grafts following the brain death of the initial recipient. World J Hepatol.

[11] Moreno EG, García GI, González-Pinto I, Gómez SR, Loinaz SC (1991). Successful reuse of a liver graft. Br J Surg.

[12] Tayar C, Karoui M, Laurent A, Hadjhamida MB, Nhieu JT, Duvoux C, Cherqui D (2006). Successful reuse of liver graft 13 years after initial transplantation. Transplantation.

[13] Moreno E, Gómez SR, Gonzalez I (1993). Neurologic complications in liver transplantation. Acta Neurol Scand.

[14] Menegaux F, Keeffe EB, Andrews BT (1994). Neurological complications of liver transplantation in adult versus pediatric patients. Transplantation.

[15] Moreno González E, Gómez R, Gonzalez Pinto I (1996). Reuse of liver grafts after early death of the first recipient. World J Surg.

[16] Laurens M, Scozzari G, Patrono D, St-Paul MC, Gugenheim J, Huet PM, Crenesse D (2006). Warm ischemia-reperfusion injury is decreased by tacrolimus in steatotic rat liver. Liver Transpl.

[17] Gómez-Lechón MJ, Serralta A, Donato MT, Jiménez N, O'connor E, Castell JV, Mir J (2004). The immunosuppressant drug FK506 prevents Fas-induced apoptosis in human hepatocytes. Biochem Pharmacol.

